# Physiological Sensing of Carbon Dioxide/Bicarbonate/pH via Cyclic Nucleotide Signaling

**DOI:** 10.3390/s110202112

**Published:** 2011-02-11

**Authors:** Jochen Buck, Lonny R. Levin

**Affiliations:** Department of Pharmacology, Weill Medical College of Cornell University, 1300 York Avenue, New York, NY 10065, USA; E-Mails: jobuck@med.cornell.edu (J.B.); llevin@med.cornell.edu (L.R.L.); Tel.: 212-746-6274 (J.B.); 212-746-6752 (L.R.L.); Fax: 212-746-6241

**Keywords:** soluble adenylyl cyclase, cAMP, second messenger, cyclic nucleotides, bicarbonate, carbon dioxide, pH

## Abstract

Carbon dioxide (CO_2_) is produced by living organisms as a byproduct of metabolism. In physiological systems, CO_2_ is unequivocally linked with bicarbonate (HCO_3_^−^) and pH via a ubiquitous family of carbonic anhydrases, and numerous biological processes are dependent upon a mechanism for sensing the level of CO_2_, HCO_3_, and/or pH. The discovery that soluble adenylyl cyclase (sAC) is directly regulated by bicarbonate provided a link between CO_2_/HCO_3_/pH chemosensing and signaling via the widely used second messenger cyclic AMP. This review summarizes the evidence that bicarbonate-regulated sAC, and additional, subsequently identified bicarbonate-regulate nucleotidyl cyclases, function as evolutionarily conserved CO_2_/HCO_3_/pH chemosensors in a wide variety of physiological systems.

## 1. Introduction

Carbon dioxide (CO_2_) and water are the major end products of energy producing pathways in living organisms (Equation (1)). As such, in non-photosynthetic organisms, CO_2_ and water represent the most fundamental catabolites.

(1) $$\text{Glucose}\;(\text{or other energy sources})+O_{2}\;\text{-----}>\;{\text{CO}}_{2}+H_{2}O$$

(2) $${\text{CO}}_{2}+H_{2}O<\text{-----}>\;H_{2}{\text{CO}}_{3}<\text{-----}>{\text{HCO}}_{3}^{-}+H^{+}$$

In unicellular organisms, CO_2_ gas can simply diffuse away, but once multicellular organisms evolved, they had to devise methods for safely dealing with CO_2_. In solution, CO_2_ combines with water to form carbonic acid (H_2_CO_3_), which dissociates to liberate a proton and a bicarbonate ion (HCO_3_^−^) (Equation (2)). CO_2_, bicarbonate and pH equilibrate on their own within minutes, but in biological systems, equilibrium is reached nearly instantaneously due to the ubiquitous presence of carbonic anhydrases [1]. This equilibrium is used to buffer pH inside cells and in intercellular fluids; for example, intracellular pH is regulated via an interplay between CO_2_ diffusion, and bicarbonate and proton transporters and/or exchangers. In mammals, and terrestrial vertebrates in general, this equilibrium is tightly controlled in two ways; the kidneys regulate the bicarbonate concentration and the breathing frequency determines the concentration of carbon dioxide. Each of these processes requires a ‘sensor,’ *i.e.*, an exquisitely sensitive and rapid way to measure the precise concentration of either CO_2_ and/or bicarbonate and/or pH and elicit an appropriate response. Many other physiological processes, in addition to diuresis and breathing rate regulation, are modulated by CO_2_ and/or bicarbonate and/or pH (*i.e*., sperm activation, blood flow, aqueous humor in the eye and cerebrospinal fluid formation), and they also require a CO_2_/HCO_3_/pH sensor. For many years, the effects of CO_2_ and pH had been ascribed to undefined chemoreceptors, and the effects of bicarbonate were traditionally thought to be mediated by changes in cellular pH [1]. In 2000, our research group demonstrated that HCO_3_^−^ directly modulates the activity of soluble adenylyl cyclase (sAC), a novel form of the enzyme generating the ubiquitous second messenger, cAMP [2], revealing that physiological CO_2_/HCO_3_/pH could be sensed via second messenger signaling.

Cyclic AMP was discovered more than 50 years ago by Earl Sutherland to act as a ‘second’ or intracellular messenger which mediated cellular responses to extracellular signals in organisms as diverse as bacteria and mammals [3]. Still, our understanding of cAMP signaling has recently undergone two transformative changes: cAMP signaling is organized into multiple, independently-regulated microdomains within a cell [4–6], and in addition to its role mediating cellular changes, cAMP can affect cellular physiology by modulating the amplitude or duration of other signaling cascades [7].

Over the decades of studying cAMP signaling in mammalian biology, this single second messenger had been implicated in a wide variety of often-contradictory physiological processes, including different aspects of metabolism, proliferation, apoptosis, differentiation, migration, development, ion transport, pH regulation, and gene expression. This seeming conundrum was finally resolved with the appreciation that cAMP acts locally within independently regulated microdomains. The microdomain model posits that cAMP is generated at distinct locations within the cell by independently regulated adenylyl cyclases [8–10], where it modulates only nearby targets, including cyclic nucleotide gated ion channels, Exchange Proteins Activated by cAMP (EPACs), or Protein Kinase A (PKA). Ultimately, the cAMP is degraded by phosphodiesterases (PDEs) which serve two functions; they act as barriers to cAMP diffusion [11,12] preventing unregulated cross-communication between microdomains [13] and the more traditionally accepted role restoring cAMP levels to their basal level terminating the signaling cascade [14]. Individual microdomains can be wholly contained within an organelle, such as the mitochondria or nucleus [9,10,15] or can be defined by A-kinase anchoring proteins (AKAPs), which tether PKA [16,17], and possibly adenylyl cyclases [18–20] and/or PDEs [21–28] to specific locations inside cells. Organization in microdomains enables this one second messenger to simultaneously mediate disparate processes throughout a cell.

There were early hints that cAMP signaling was compartmentalized within a cell; for example, distinct hormones which have the same effects on cAMP levels in bladder epithelial cells do not have the same effect on osmotic water flow [29]. But a need for independently-regulated cAMP microdomains was best demonstrated in cardiomyocytes, where it was observed that two hormones, which both functioned via cAMP, elicited completely different responses [30]. Modern FRET-based [31–33] and biophysical methods [34,35] that enable measuring cAMP concentrations *in situ* revealed that cAMP levels are not uniform within cells (reviewed in [4,5]). The microdomain organization of cAMP signaling was definitively confirmed by the demonstration of independently regulated, membrane-proximal cAMP microdomains in neurons [36] and cardiomyocytes [37]; by the demonstration of the role of AKAPs [16,17]; and by the unique functions of artificial, localized production of second messenger within distinct subcellular compartments [38–40]. Among the implications for a locally acting second messenger is the realization that changes in cAMP levels do not have to be large (or even detectable in a whole cell context) to be physiologically relevant; meaningful cAMP fluctuations within a microdomain could be insignificant compared to the total cAMP content of a cell. Thus, even for a cAMP-mediated process, measuring a cAMP rise may prove difficult. The microdomain organization of signaling seems to be true for both cAMP and the other second messenger cyclic nucleotide, cGMP; in cultured hippocampal neurons, localized cAMP was shown to be essential for axonal determination while compartmentalized cGMP defined dendrites [41].

**Figure 1.**
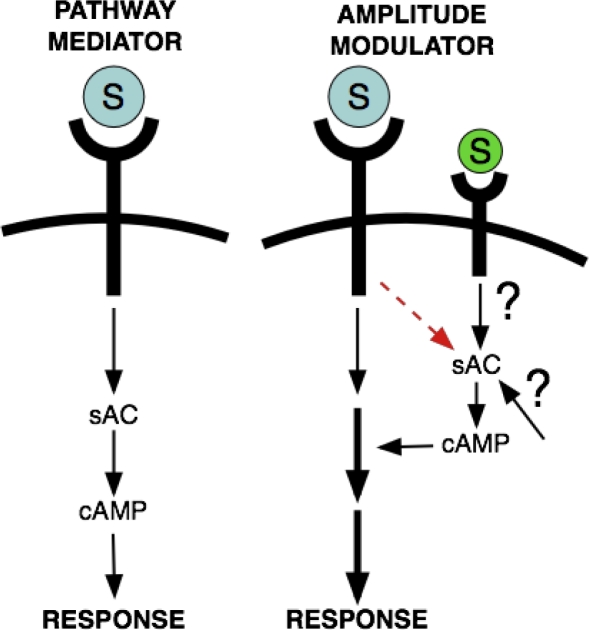
**Mediator *vs*. Modulator.** In the mediator pathway, sAC generated cAMP functions as part of “bucket brigade” being both necessary and sufficient to elicit a response. In the modulator pathway, where sAC-generated cAMP controls the magnitude or duration of a response, sAC activity could be regulated by a distinct extracellular signal, by intracellular signals (*i.e*., CO_2_/HCO_3_^−^/pHi), or as a secondary effect of the primary signal mediating the cellular response (red arrow).

The concept of cAMP as an amplitude or frequency modulator of other signaling pathways derives from an idea posited 15 years ago by Ravi Iyengar [7]. In addition to its role as a signal mediator (Iyengar referred to this role as functioning as part of a “bucket-brigade” where cAMP is both necessary and sufficient to elicit a response), he suggested that cAMP might be functioning as a “gate” to regulate information flow through distinct signaling pathways. In his “gating” model, cAMP served a permissive role, turning a pathway on or off. Our studies of sAC have confirmed and extended this model for cAMP function; our studies identified a role for sAC-generated cAMP functioning like a rheostat, modulating intensity or frequency of a signaling pathway (Figure 1).

As described in more detail below, sAC is responsible for CO_2_-dependent regulation of oxidative phosphorylation in mitochondria [42]. In performing this role, sAC-generated cAMP does not elicit a response on its own, but functions as an ‘amplitude modulator;’ it alters the rate of ATP production dependent upon the amount of metabolically generated CO_2_. sAC also has modulatory functions in the CO_2_-dependent regulation of beat frequency of cilia in airway epithelia [43] and the HCO_3_-induced activation of sperm motility [44–46]; in both cases, sAC-generated cAMP alters the frequency of an already existing beating response (in cilia or flagella, respectively). Interestingly, sAC in sperm exhibits both types of functionalities; sAC-generated cAMP acts as a ‘frequency modulator’ to control the rate of flagellar beating for hyperactivated motility, but it also acts as an “on-off” pathway mediator, initiating swimming and the process of capacitation, the developmental program needed to enable sperm to penetrate and fertilize an egg [44,45,47]. Other examples where cAMP seems to serve a “gating” function included growth factor activation of the MAP Kinase pathway [48], long-range patterning induced by the diffusible morphogen, Sonic Hedgehog [49–51], long-term potentiation evoked by repeated stimulation in hippocampal CA1 region [52], and neurotrophin-dependent survival and growth of neurons [53]. Interestingly, these processes may also involve sAC [54–58].

‘Amplitude or frequency modulation’ provides a mechanism for cells to fine tune responses to a signal such that more (or less) signal is required to provide a consistent or maintained response. This property is particularly useful for a gradient morphogen or any diffusible signal that induces directional movement, such as a neuronal guidance cue, where a cell responds by moving up (or down) a concentration gradient.

## 2. Discovery of sAC and Regulation by Bicarbonate

G protein regulated, transmembrane adenylyl cyclases (tmACs) mediate intracellular changes due to extracellular signals such as hormones and neurotransmitters binding to G protein coupled receptors (GPCRs); for a long time, these were thought to be the predominant (if not only) sources of cAMP in higher eukaryotes. In 1999, our laboratory purified and cloned mammalian soluble adenylyl cyclase (sAC) [59] defining a unique signaling enzyme (Table 1; Reviewed in [60]). sAC is more closely related to (cyano)bacterial ACs than to tmACs or other metazoan cyclases providing a link between prokaryotic and eukaryotic signal transduction mechanisms. Isoform diversity for tmACs is generated via nine distinct genes; whereas for mammalian sAC, a single gene is alternatively spliced [61,62] and uses multiple promoters [63]. Unlike tmACs, sACs are not transmembrane proteins and are found distributed throughout the cytoplasm and in specific organelles [9,10,15] where they are thought to be the source of second messenger mediating the intracellular functions of cAMP [8,15]. As stated above, tmACs are directly modulated by heterotrimeric G proteins which transduce extracellular signals into intracellular cAMP changes. In contrast, sAC isoforms are insensitive to heterotrimeric G proteins [59] but are instead regulated by intracellular signals, including bicarbonate [2,64–67], calcium [68,69], and ATP [69].

Structurally, sAC and tmACs are quite similar [70]; both sAC [70] and tmACs [71] are active as dimers of two catalytic (C) units (Reviewed in [60,72]). However, structures (to a resolution of 1.9 Å) of various complexes of a bicarbonate- and calcium-regulated bacterial sAC-like cyclase with different substrate analogs provide a rationale for sAC-like cyclases’ insensitivity to heterotrimeric G proteins and their lower affinity for substrate ATP. These structures also reveal how calcium increases sAC-like cyclases’ affinity for ATP, and how bicarbonate stimulates catalytic rate. Bicarbonate regulation is conserved in sAC-like cyclases throughout evolution [2,73–76] as well as in yeast adenylyl cyclases [77–79] and a number of transmembrane (*i.e*., receptor-type) guanylyl cyclases [80–83]; thus, bicarbonate regulation of cyclic nucleotide synthesis is poised to be an evolutionarily conserved mechanism for physiological sensing CO_2_/HCO_3_/pH. In this review, we focus specifically on the functions of sAC (and other bicarbonate-regulated cyclases) where it functions as a physiological CO_2_/HCO_3_/pH chemosensor. Broader reviews, describing the various functions of mammalian sAC [84] and the variety of physiological CO_2_/HCO_3_/pH chemosensors [58], have recently been published.

## 3. Physiological CO_2_/HCO_3_/pH Chemosensing via sAC

### 3.1. Bicarbonate Activation of Sperm

Morphologically mature epididymal sperm do not have the “capacity” to fertilize an egg [85]. They acquire fertilization-competence during ejaculation and transit through the female reproductive tract. Upon ejaculation, sperm acquire flagellar motility (*i.e*., swim) and begin a poorly defined maturation process called capacitation. Capacitation continues inside the female reproductive tract, where it includes hyperactivation of flagellar motility and attaining the ability to perforate the egg’s zona pellucida via the acrosome reaction. These events lead to binding and fusion to the egg’s plasma membrane and fertilization. At least two of these stages, motility and capacitation, are induced by bicarbonate [86–89] and dependent upon cAMP signaling [89–92].

We originally purified sAC from testis [59] and sAC mRNA is highly expressed in male germ cells [93]. At least two isoforms of sAC are present in male germ cells [44]: a 187 kDa protein (“full length”, or sAC_fl_) and a shorter, 53 kDa variant (“truncated”, or sAC_t_) [59]. sAC_t_ has an approximately ten times higher specific activity than sAC_fl_ [94], and while both are found in testis and sperm [44,95,96], sAC_t_ appears to be responsible for the majority of cAMP production in mature sperm [44,45,47,97]. We (and others) demonstrated that the effects of bicarbonate on sperm are directly mediated by sAC [44,45,47,97]. Specifically, both motility [44,47,97] and capacitation [44,45] are abrogated in sAC knockout mice and by the sAC-specific pharmacological inhibitor, KH7 [44].

### 3.2. pH Sensing

Prior to ejaculation, sperm are stored in the cauda epididymis where they are maintained in a quiescent state by an acidic pH of 6.5–6.8 and a low bicarbonate concentration of 2–7 mM (compared to 25 mM in serum, prostate and other bodily fluids) [98]. In 2003, we demonstrated that sAC functions as a pH sensor in the clear cells of the epididymis to ensure that the luminal pH and bicarbonate concentration remain low [99]. sAC is highly expressed in clear cells, and apical membrane accumulation of the proton pumping vacuolar ATPase (V-ATPase) is triggered by a sAC-dependent rise in cAMP in response to alkaline luminal pH. The apical mobilization of the V-ATPase is also dependent upon carbonic anhydrase (CA), the enzyme responsible for the nearly instantaneous equilibration of pH and HCO_3_^−^, presumably facilitating sAC activation by bicarbonate in response to elevated pH.

sAC [76], CA [100], and V-ATPase [101,102] are also instrumental in regulating the recovery from alkalotic challenge in the dogfish shark. In the shark gill, which is the main acid-base sensing organ of this ancient vertebrate, alkalotic stress induces a sAC- and CA-dependent translocation of V-ATPase into the basolateral membrane of the gill. The V-ATPase then pumps protons back into the body to counter the systemic alkalosis. Additionally, sAC forms a complex with the V-ATPase in acid-base transporting intercalated cells in mammalian kidney [103], and sAC, CA and V-ATPase are postulated to mediate proton secretion from acid (A-type) secreting cells into the renal collecting duct [104]. Thus, sAC, CA and V-ATPase seem to form a functional unit for sensing, and responding to, alterations in pH [105]. Interestingly, the sAC-CA-V-ATPase mechanism is capable of moving the proton transporter to wherever it is needed; *i.e.*, the V-ATPase translocates to the apical membranes in clear cells of the epididymis and A-type cells of the renal collecting duct while it moves to the basolateral membrane in the shark gill. Because sAC, CA, and V-ATPase are evolutionarily ancient, it is tempting to hypothesize that this functional unit for sensing pH and moving protons to correct pH imbalances will be found widely utilized throughout biology.

### 3.3. CO_2_ Regulation of Beating Frequency of Cilia on Airway Epithelia

Airway epithelial cells express motile cilia that are important for innate host defense; the beat of the cilia removes the mucous layer clearing toxins, pathogens, allergens, and debris [106]. To accomplish this feat, cilia beat faster during exhalation relative to inhalation. Exhaled breath has higher CO_2_ than inspired air. sAC ‘senses’ this elevated CO_2_, and sAC-generated cAMP activates PKA which increases the frequency of ciliary beating during exhalation [43]. This represents an example where sAC-generated cAMP acts as a pathway modulator; CO_2_ chemosensing via sAC controls the rate of ciliary beating, not whether or not the cilia beat.

### 3.4. Krebs Cycle Generated CO_2_ Regulates the Rate of Oxidative Phosphorylation

sAC resides inside mitochondria [9,15,107] where it coordinates the rate of ATP production via oxidative phosphorylation (OXPHOS) with nutritional availability. Mitochondrial sAC activity is stimulated by Krebs Cycle-generated CO_2_ in a carbonic anhydrase dependent manner [15]. CO_2_/HCO_3_^−^ stimulation of sAC activates intramitochondrial PKA which phosphorylates Complex IV of the electron transport chain, increasing its rate and capacity to handle electrons. Because the electrons feeding the electron transport chain also originate from the Krebs Cycle, this mitochondrial CO_2_-sAC-cAMP-PKA pathway couples nutrient utilization (*i.e*., Krebs Cycle activity) to ATP production. Once again, this pathway does not turn on or off the electron transport chain, it simply modulates the rate of ATP generation to ensure optimal utilization of electrons.

### 3.5. Physiological Processes Dependent upon CO_2_/HCO_3_/pH which May Involve sAC

There are a number of physiological processes where CO_2_/HCO_3_/pH chemosensing is known to play a role, but the chemosensor is not yet identified. Some are even thought to employ cAMP as a second messenger, but involvement of sAC has yet been demonstrated. For example, bone resorption by osteoclasts is thought to be mediated via V-ATPase dependent proton pumping [108]. And while sAC seems to regulate growth and differentiation of osteoclasts [109], there is only circumstantial evidence that sAC plays a role in bone formation. Human sAC was identified as a locus for absorptive hypercalciuria (AH), a kidney stone-forming condition frequently complicated by bone loss [110], and polymorphisms in the human sAC locus are associated with phenotypic variations in bone mineral density [111].

Cerebrospinal fluid formation (CSF) by the choroid plexus and aqueous humor formation by ocular ciliary processes are dependent on bicarbonate [112,113]. In ciliary processes and choroid plexus transport systems, carbonic anhydrase inhibitors decrease fluid secretion [114], and carbonic anhydrase inhibitors can be used to treat glaucoma, a fluid secretion defect in the eye. sAC seems to be present in choroid plexus [2] and in ciliary processes [115], but as yet, there have been no functional studies linking sAC to either process.

Partial CO_2_ pressure (PCO_2_) is the main determinant of ventilation rate [1]. Elevations of PCO_2_ increase breathing frequency, while decreased PCO_2_ slows breathing frequency. These rate changes are mediated by peripheral and central chemoreceptors which monitor changes in arterial PO_2_ and PCO_2_ blood gases. The peripheral chemoreceptors are in the carotid and aortic bodies, and their actions have long been thought to be due to alterations in intracellular pH (pH_i_). However, studies in the chemosensitive (glomus) cells of the carotid body reveal a direct role for CO_2_, independent of pH_i_ [116]. These studies also demonstrated that elevations in PCO_2_ elicited an increase in glomus cell cAMP leading the authors to suggest involvement of sAC. PCO_2_ also plays a role in regulating blood flow. Blood flow is tightly coupled to tissue metabolism [1]; cerebral arterioles dilate in response to increases in metabolic activity, and CO_2_, protons, and adenosine function as vasodilators by relaxing smooth muscles. Cerebral arterioles are exquisitely sensitive to the vasodilatory action of PCO_2_; however, the molecular nature of the vascular PCO_2_ receptor is unknown. Interestingly, cAMP was postulated to be downstream of the CO_2_ signal [117], but once again, functional studies assessing sAC’s role in sensing circulating PCO_2_ have not yet been performed.

## 4. Evolutionary Conservation of Physiological CO_2_/HCO_3_/pH Chemosensing via Nucleotidyl Cyclases

### 4.1. Fungal Adenylyl Cyclases Integrate CO_2_ Sensing with cAMP Signaling and Virulence

The CO_2_ concentration in mammals (5%) is more than 150 fold higher than in atmospheric air (0.033%). We identified this difference in CO_2_ as a physiological signal inducing the yeast-to-hyphal transition essential for virulence of the fungal pathogen, *Candida albicans* [77]. The *C. albicans* adenylyl cyclase (AC) is directly stimulated by HCO_3_, and it is responsible for ‘sensing’ in a carbonic anhydrase dependent manner, the elevated CO_2_ inside infected hosts. CO_2_/HCO_3_ regulation of cAMP synthesis is conserved in other fungi. In the fungal pathogen *Cryptococcus neoformans*, capsule formation is essential for evading host immune detection. Once again, the signal inducing capsule formation is the higher CO_2_ concentration inside the infected host, and the *C. neoformans* cyclase serves as the pathogen’s CO_2_/HCO_3_ chemosensor [78].

### 4.2. CO_2_ Chemosensing via cGMP Signaling

The nematode *Caenorhabditis elegans* also senses environmental CO_2_. In contrast to many parasitic nematodes, the free-living *C. elegans* avoids CO_2_ [118,119], and this response is dependent upon expression of the GCY-9 receptor-type guanylyl cyclase (along with cyclic nucleotide gated ion channels) in the CO_2_ chemosensing (BAG) sensory neurons [120]. Interestingly, *C. elegans* also avoid high levels (in excess of 12%) of oxygen; this response is mediated by a distinct subset of sensory neurons, but it also involves a receptor-type guanylyl cyclase (GCY-35) and cyclic nucleotide gated channels [121].

The fruit fly *Drosophila melanogaster* also avoids environmental CO_2_, and while this response requires two GPCR-like olfactory receptors [122], involvement of a cyclic nucleotide second messenger remains unclear [123]. In mammals, the question of sensing environmental CO_2_ via cyclic nucleotides also remains unresolved. A particular subset of olfactory neurons in mice seemed to be capable of sensing concentrations of CO_2_ approaching environmental levels [124]. These neurons express a transmembrane guanylyl cyclase, GC-D, which was subsequently demonstrated to be bicarbonate regulated [82,83]. A second transmembrane guanylyl cyclase, GC-G, which is also found in the olfactory system, has also been demonstrated to be directly modulated by bicarbonate [80]. Sensory detection of environmental CO_2_ in a number of organisms was recently reviewed in [125]. While these findings cement the linkage between CO_2_/HCO_3_/pH chemosensing and cyclic nucleotide signal transduction, their physiological significance remains unknown.

## 5. Summary and Future Trends

In physiological systems, CO_2_, HCO_3_^−^, and pH are intimately linked via carbonic anhydrases, and a variety of biological processes, in mammals and throughout evolution, depend upon a CO_2_/HCO_3_/pH chemosensor. Bicarbonate-regulated sAC, which links intracellular CO_2_, HCO_3_^−^, and/or pH levels with cAMP signal transduction, serves as the CO_2_/HCO_3_/pH chemosensor in at least a subset of these processes. The future will reveal whether other CO_2_/HCO_3_/pH chemosensing functions are also mediated by sAC. Bicarbonate regulation is observed in other mammalian nucleotidyl cyclases and in adenylyl cyclases across evolution implying that cyclic nucleotide signaling is an evolutionarily conserved mechanism for CO_2_/HCO_3_/pH chemosensing.

## Figures and Tables

**Table 1.** The two distinct classes of mammalian adenylyl cyclase.

	**sAC**	**tmACs**
**Evolutionary relatedness**	(Cyano)bacteria	‘First’ Appearance: Dictyostelium
**Isoform variability**	One gene with multiple splice variants and an alternative start site	Nine distinct genes
**Tissue distribution**	Ubiquitous	Ubiquitous
**Subcellular localization**	Cytoplasm, nucleus, mitochondria, centrioles, mitotic spindle, mid-body	Plasma membrane
**Physiological Modulators**	Bicarbonate, calcium, & ATP	G proteins & other 2nd messengers
**Functions**	HCO_3_^−^ sensing in sperm pH sensing in acid/base sensing epithelia CO_2_ sensing in airway cilia and mitochondria	Intercellular signaling (*i.e*., hormones, neurotransmitters, odorants)
